# Community-Based 4-Level Intervention Targeting Depression and Suicidal Behavior in Europe: Protocol for an Implementation Project

**DOI:** 10.2196/64218

**Published:** 2025-01-10

**Authors:** Katharina Schnitzspahn, Kahar Abdulla, Ella Arensman, Chantal Van Audenhove, Rainer Mere, Victor Pérez Sola, Merike Sisask, András Székely, Piotr Toczyski, Ulrich Hegerl

**Affiliations:** 1 European Alliance Against Depression e.V. Leipzig Germany; 2 Ruhr University Bochum Bochum Germany; 3 University College Cork National Suicide Research Foundation Cork Ireland; 4 Australian Institute for Suicide Research and Prevention Griffith University Brisbane Australia; 5 LUCAS–Centre for Care Research and Consultancy KU Leuven Leuven Belgium; 6 Estonian-Swedish Mental Health and Suicidology Institute Tallinn Estonia; 7 School of Governance, Law and Society (SOGOLAS) Tallinn University Tallinn Estonia; 8 Centro de Investigación Biomédica en Red de Salud Mental Madrid Spain; 9 Végeken Egészséglélektani Alapítvány Budapest Hungary; 10 Maria Grzegorzewska University Warsaw Poland; 11 Goethe University Frankfurt Germany

**Keywords:** depression, suicide, mental health, European Alliance Against Depression, EAAD, 4-level community-based intervention, iFightDepression, cognitive behavioral therapy, mHealth

## Abstract

**Background:**

The community-based, 4-level intervention of the European Alliance Against Depression (EAAD) is simultaneously addressing depression and suicidal behavior. Intervention activities target primary care health professionals (level 1), the general public (level 2), community facilitators (level 3), and patients and their relatives (level 4). Activities comprise the digital iFightDepression tool, a guided self-management tool based on cognitive behavioral therapy.

**Objective:**

This study aimed to present the European Union–cofunded EAAD-Best study protocol, aiming at the implementation, dissemination, and evaluation of the 4-level intervention and the iFightDepression tool in several countries across Europe.

**Methods:**

The 4-level intervention has been implemented for the first time in Bulgaria, Estonia, Greece, and Poland. In 3 countries that have already implemented the 4-level intervention (Hungary, Ireland, and Spain), activities have been extended to new regions. In addition, the nationwide uptake of the iFightDepression tool by patients with depression has been promoted in all mentioned countries and Italy.

**Results:**

To evaluate the implementation of the 4-level intervention and the iFightDepression tool, data related to the process, output, and outcome were collected between 2022 and 2024. Data processing and analyses started in 2023. Analyses are expected to be completed in 2024. Results are expected to be published in 2025.

**Conclusions:**

This paper informs researchers, practitioners, and stakeholders on how to implement best practices in mental health promotion and evaluate their effectiveness.

**International Registered Report Identifier (IRRID):**

DERR1-10.2196/64218

## Introduction

Depression is one of the most prevalent, severe, and undertreated mental disorders [1]. Having a diagnosis of depression is associated with a reduction in life expectancy of around 10 years or more [2]. Contributing to this is the fact that depression increases the risk of another important mental health issue—suicidal behavior [3]. Approximately 703,000 people die by suicide every year [4] and the number of attempted suicides is considerably higher and rising [5]. The vast majority of completed suicides occur in the context of depressive disorders and other mental illnesses [2].

Due to the numerous factors contributing to suicidal acts, the best approach to address suicidal behavior is multifaceted interventions [6] that have been shown to be most effective [7]. Such multilevel intervention programs have been developed in different countries and either focus exclusively on suicidal behavior or simultaneously aim to improve care for people with depression [6]. A recent systematic review concluded that the most promising multifaceted intervention to prevent suicide is the 4-level intervention approach by the European Alliance Against Depression (EAAD) [8]. The authors reviewed 56 publications (describing 47 unique studies) in Linskens et al [8] on community-based or population-level suicide prevention strategies. EAAD’s 4-level intervention offered the most evidence for its effectiveness, while evidence for other single- or multistrategy interventions was either unclear, inconsistent, or lacking. The authors, therefore, suggest that further randomized or observational studies are needed. The EAAD-Best project introduced in this paper will contribute to this identified research need.

**Figure 1:**
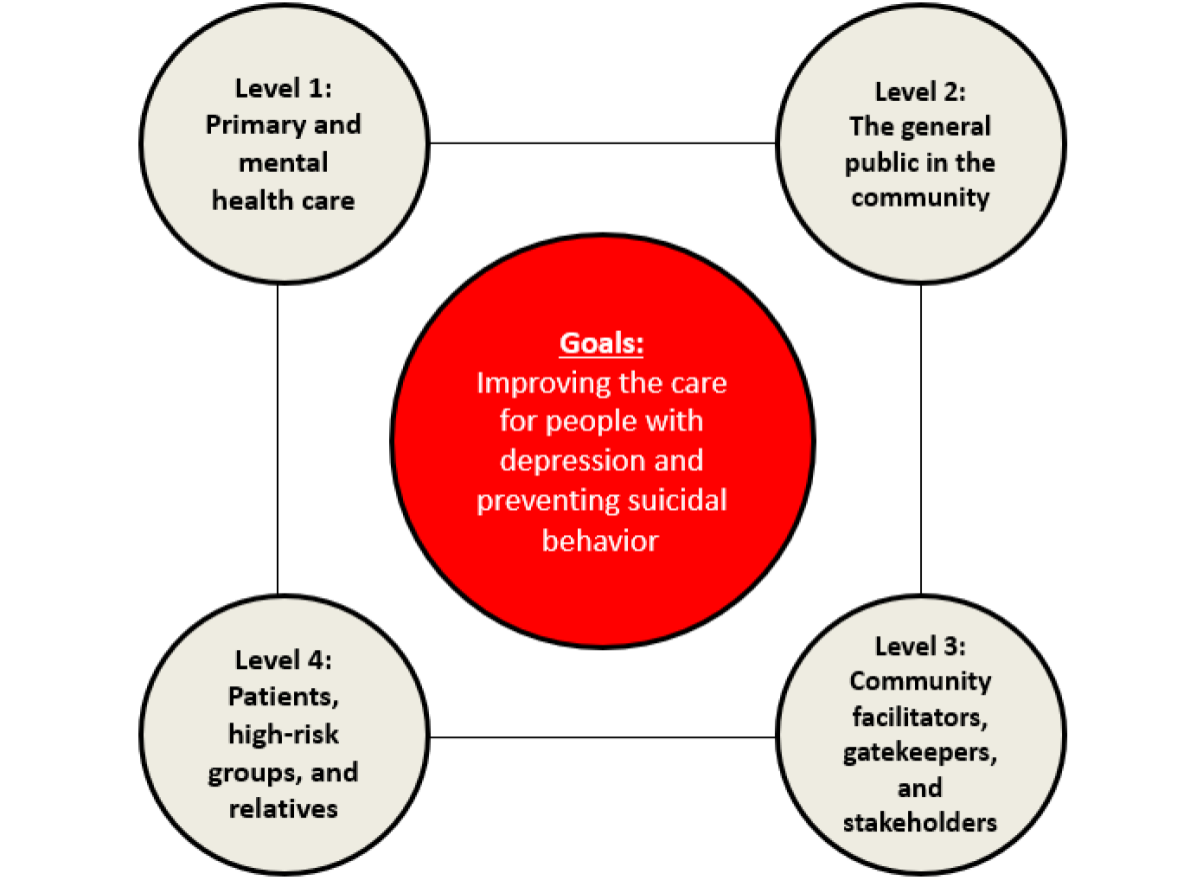
European Alliance Against Depression’s community-based, 4-level intervention.

The 4-level intervention has been implemented in local communities and simultaneously runs activities at 4 intervention levels (Figure 1).

- Level 1: Interventions aim to improve the capacity of general practitioners (GPs) and mental health care professionals (MHCPs) regarding the management of depression and suicidality. This is achieved by training sessions focusing on early detection, diagnosis, and treatment.
- Level 2: Interventions target the broader public and aim to raise awareness, increase knowledge on depression and suicide, reduce stigma, and improve help-seeking behavior. This is achieved through a public awareness campaign. It emphasizes the need for treatment, the availability of effective treatment options, and support services. Key messages are (1) depression is a real disease; (2) depression can affect anyone; (3) depression has many faces; and (4) depression can be treated.
- Level 3: Interventions aim to improve the capacity of community facilitators and gatekeepers concerning the recognition and handling of depression and suicide risk. Community facilitators and gatekeepers are nonmedical professionals who have a public and social function in society (geriatric caregivers, priests, pharmacists, police, journalists, counselors, midwives, teachers, social workers, managers, and company physicians). They frequently interact with people who are experiencing depression or suicidal ideation and are therefore well positioned to provide general preventive and supportive services. Journalists are informed about the risk of triggering the so-called Werther effect (copycat suicides) and are offered a media guide with recommendations on how to report and not to report about suicidal acts in order to reduce the risk of unwanted effects of media coverage. Capacity building is achieved through train-the-trainer sessions adapted to the specific needs of (subgroups of) community facilitators.
- Level 4: Interventions aim to support patients who are depressed and/or suicidal and their relatives by supporting self-help (eg, implementation of self-help groups and promoting the iFightDepression tool) and providing information about depression, suicidal behavior, and treatments. Identifying locations where people frequently take or attempt to take their lives in a certain region and starting activities to secure these places is also an element within the 4-level intervention.

Implementation of the 4-level intervention is supported by the EAAD coordination center that developed an implementation guide, a catalog of step-by-step recommendations (eg, how to organize a press conference and how to recruit GPs), and has accumulated a broad range of intervention and evaluation materials over the last years. Many materials have already been translated into various languages including the iFightDepression awareness website [9]. This website provides information about depression, its causes, symptoms, and treatments and is publicly accessible. The effectiveness of the 4-level intervention to reduce suicidal behavior was demonstrated by several studies in different countries, although not all evaluated community-based interventions showed the expected positive effects [10-13].

Within the 4-level intervention, the web-based iFightDepression tool is an important intervention component that can help reduce the treatment gap concerning psychotherapeutic interventions [14]. It is based on the principles of cognitive behavioral therapy and supports the self-management of people with mild-to-moderate forms of depression. Access and guidance to the tool are provided by health care professionals. The safety [15] of the tool and its efficacy [14,16] have been assessed for mild-to-moderate symptoms of depression in a randomized controlled trial with an active control group [14] and a study with a treatment-as-usual control group [16]. It is possible that patients with severe depression can benefit from digital interventions [17], but the risk of failing to use the intervention due to motivational problems that are often part of major depression also seems more likely and could cause further frustration and self-criticism. The recommended use of the iFightDepression tool is therefore currently limited to the patient groups for which solid evidence is available, but as research progresses, this might be extended.

The 4-level intervention has been successfully adapted to different cultures and health care systems in more than 120 regions within and outside of Europe [18]. Important lessons that have been learned with growing experience are summarized as follows:

1. Targeting optimized care of depression and suicide prevention simultaneously is highly cost-effective and sensible for several reasons. Both are highly important mental health issues and depression is one of the key causal factors of suicidal behavior. Suicide-preventive measures unrelated to depression, such as access to lethal means, can easily be integrated in the 4-level intervention program. Given the high prevalence of depression, a public campaign on depression is likely to have a greater resonance than an exclusive suicide prevention campaign. The latter may further bear the risk of reducing the threshold for suicide due to the normalization of suicidal behavior or unfavorable secondary reporting, for example, in social media.
2. Being simultaneously active at all 4 intervention levels creates synergistic and catalytic effects [19]. An example of a synergistic effect is that the public health campaigns (level 2) not only increase awareness and knowledge in the general population but also increase the motivation of GPs and gatekeepers to participate in the training offered (levels 1 and 3). An example of a catalytic effect is the observed improvement of intersectoral cooperation between health professionals in communities participating in the 4-level intervention, an effect that has not directly been targeted by the activities [19].
3. Depending on the culture and health care system, intervention activities must find the right balance between bottom-up and top-down approaches. A bottom-up approach creates an ownership feeling in the community and is helpful to achieve sustainability. However, in countries with a less developed civil society and a more hierarchical political culture, more top-down support is required.
4. The pharmaceutical industry should not be involved in funding and implementation activities as this may reduce credibility and create conflicts of interest.
5. The implementation of the 4-level intervention in an initial model region raises interest in other regions, causing them to follow suit. The creation of a learning network of all implementing regions, which meet 2 or 3 times per year to exchange experiences and support each other, has proven useful.

Taking these and other lessons learned into account, the EAAD-Best project (full title: “Adapting and implementing EAAD’s best practice model to improve depression care and prevent suicidal behavior in Europe”) was set up and received funding from the Third Health Program (HP-PJ-2020) of the European Union (EU). As the title describes, the overall objective was the improvement of depression care and suicide prevention in Europe given the strongly identified need for correct diagnoses of depression and suicidality as well as effective, evidence-based interventions. The project ran from April 2021 to March 2024 and allowed us to further adapt the 4-level intervention to new contexts and evaluate its effectiveness to add to the sparse database available for multifaceted suicide prevention interventions [8]. This paper aims to introduce the EAAD-Best project as a best practice model for the implementation of mental health interventions and their evaluation.

## Methods

### Goals of EAAD-Best

The central goals of EAAD-Best were the dissemination of the 4-level intervention to further regions in Europe and the nationwide uptake of the digital iFightDepression-guided self-management tool. These goals were achieved by:

1. The implementation of the 4-level intervention in model regions of EU countries in which the intervention had not yet been established (ie, implementation countries).
2. The dissemination of the 4-level intervention to new regions in EU countries in which this approach had already been implemented in a model region (ie, transfer countries).
3. The nationwide promotion the iFightDepression tool in participating countries and recruiting health professionals as guides who offer the tool to their patients.

### Overview of EAAD-Best Activities

#### Overview

**Figure 2:**
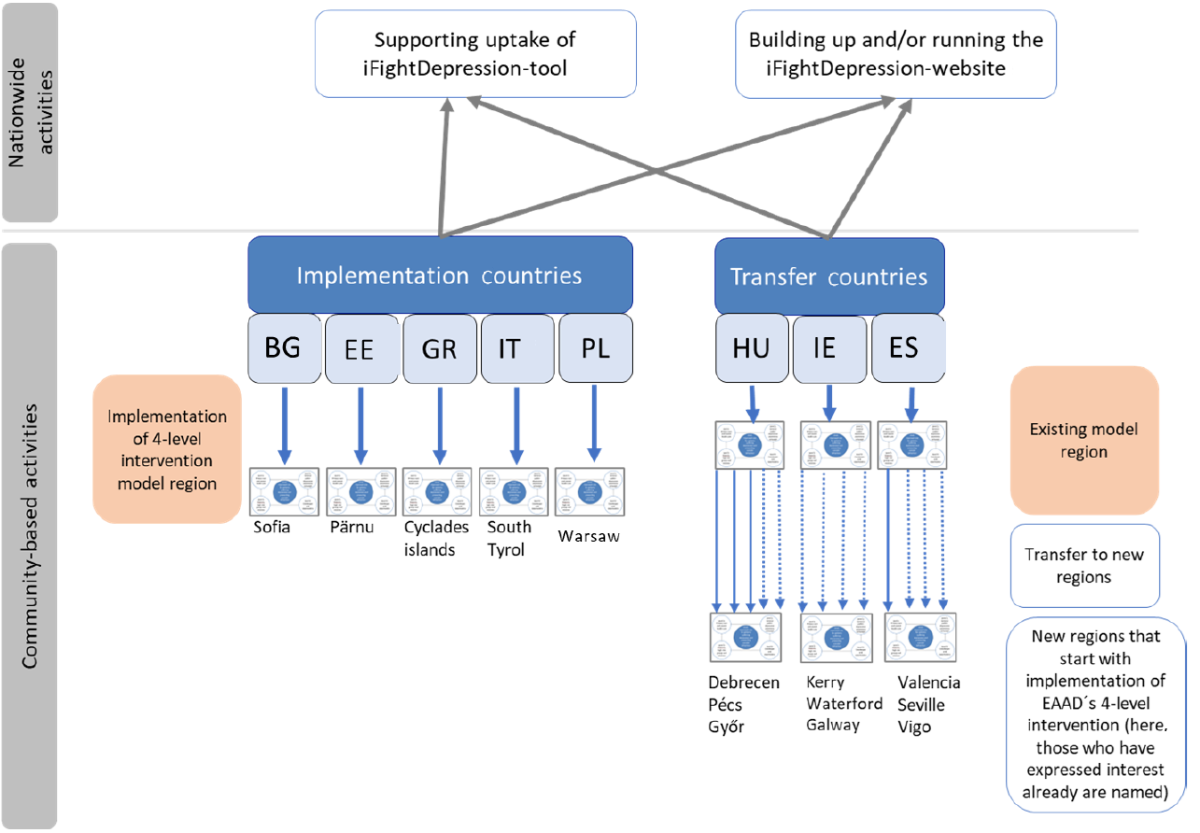
Basic structure of activities within EAAD-Best. *Due to the dropout of the Italian consortium member, Italy is no longer a full-fledged implementation country but focuses on the implementation of the iFightDepression tool and awareness website. BG: Bulgaria; EAAD: European Alliance Against Depression; EE: Estonia; ES: Spain; GR: Greece; HU: Hungary; IE: Ireland; IT: Italy; PL: Poland.

The basic structure of EAAD-Best activities is illustrated in Figure 2.

The participating countries were chosen to represent the different regions of the EU. They differ not only in population and gross national income per inhabitant but also in their health care systems and national approaches to mental health, including the regional health care system facilities for patients with depression.

#### Implementation Countries

In 4 countries, the 4-level intervention was implemented for the first time: Bulgaria, Poland, Estonia, and Greece. Italy was named as another implementation country in the initial EAAD-Best proposal, but the Italian partner changed during the project duration and therefore could only participate in the implementation of the nationwide uptake of the iFightDepression tool.

Model regions were Sofia (Bulgaria), Warsaw central district (Poland), Pärnu (Estonia), and 2 regions in Greece (ie, North Attica and Cyclades Islands). They were chosen based on accessibility for project partners and identified needs.

#### Transfer Countries

A total of 3 countries (Ireland, Spain, and Hungary) with previous experiences with the 4-level intervention and existing model regions offered the intervention to new regions. Ireland extended activities to Kerry and Cork. Spain targeted Valencia, Seville, Alcala la Real, and Vic, and Hungary focused on Szeged, Békéscsaba, Szentendre, and Baja. The regions were chosen based on accessibility for project partners, identified needs, and expressed interest.

The iFightDepression tool was promoted nationwide in all participating countries. It targets persons with mild-to-moderate forms of depression. Specific inclusion criteria are (1) age ≥15 years; (2) access to a computer, an internet connection, and an email account; and (3) mild-to-moderate forms of depression. Exclusion criteria are (1) age <15 years, (2) severe depression, (3) acute suicidality, and (4) current substance abuse.

### Implementation Processes in EAAD-Best

#### Initial Implementation of the 4-Level Concept in Implementation Countries

The process of implementing the 4-level intervention in implementation countries can be broken down into 3 steps:

- Step 1: Planning, design, and strategy
- Step 2: Preparation
- Step 3: Implementation

**Figure 3:**
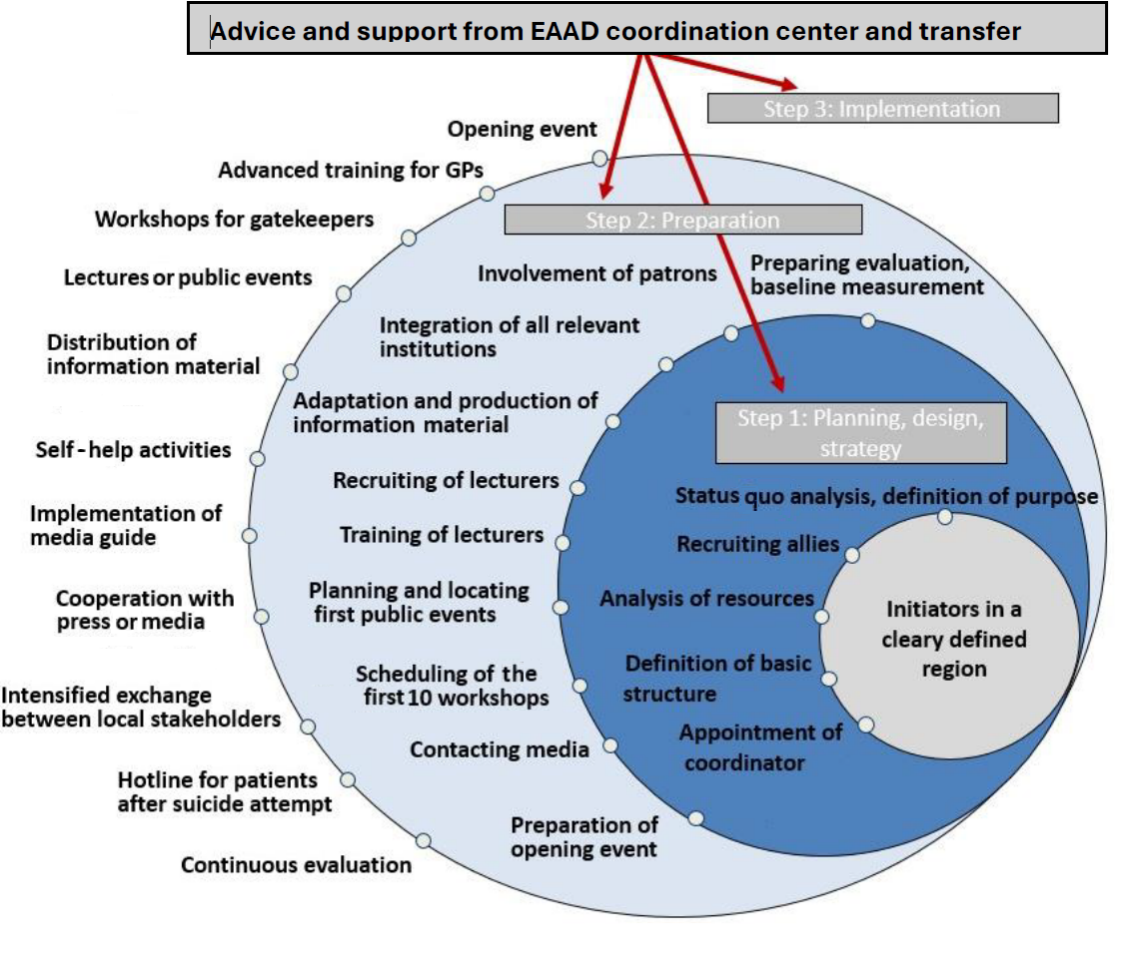
Foreseen activities for planning, preparing, and implementing the European Alliance Against Depression’s 4-level concept in the implementation countries. EAAD: European Alliance Against Depression; GP: general practitioner.

Within each step, there is a range of tasks and actions to consider (Figure 3).

#### Step 1: Planning, Design, and Strategy

Status quo analyses in the model regions identify relevant stakeholders and institutions as well as potential gaps and limitations of the local care system (ie, “As Is” state). Next, the “To Be” state is defined (ie, what the local alliance seeks to achieve). Specific objectives can be derived by comparing the “As Is” state with the “To Be” state.

The identified network of stakeholders should be broad and include existing local health care initiatives and personal links and partnerships. Stakeholders or potential sponsors should be involved in the preparation phase to increase their identification with the alliance.

Next, the alliance initiator is identified. It can be a person (eg, a psychiatrist), a small organization (eg, a self-help group), or an institution (eg, a psychiatric hospital). They should decide about the basic structure of the local alliance (eg, will it be part of an existing organization or will a nongovernmental organization be founded?). A small steering group (about 2-5 people) for leadership of the alliance should be formed and a local coordinator should be appointed.

#### Step 2: Preparation

In the preparation phase, the alliance is being formed and the interested institutions and stakeholders become integrated. Regular meetings are crucial, as most questions and fundamental decisions occur in this stage.

Materials required for activities on all 4 intervention levels must be translated and culturally adapted to regional needs (eg, public relation campaign materials, level-1 and -3 training materials, and iFightDepression awareness website). Templates are provided by the EAAD coordination center.

The professional groups that will receive training as part of intervention activities on levels 1 and 3 are defined and recruited as lecturers to run training sessions for other GPs, MHCPs, and community facilitators or gatekeepers. The first of these train-the-trainer training sessions take place and further ones are scheduled, so that respective dates can be announced at the opening ceremony and activities can start immediately afterward. A train-the-trainer information package including the referral of patients to the iFightDepression tool is provided by the EAAD.

A large public opening ceremony and further public events are prepared. Many types of public events are possible, such as public talks, podium discussions, theme nights, information booths, school projects, art exhibitions, or religious services. A range of events that are best suited to maximize contact with the target group is advisable.

A prominent and broadly respected person in the region should be involved as a patron. They do not need to have any connection with depression or suicide but should be willing to publicly support the alliance and its aims. A prominent patron will help gain support and cooperation from many partners, including the media.

The media are contacted actively, for example, by offering press releases on events or interviews on depression-related topics to journalists. The use of social media is also advised. It is recommended to appoint a person within the alliance responsible for all social media activities and “marketing” of the campaign.

#### Step 3: Implementation

During the implementation phase, different activities will be run simultaneously at the 4 levels. EAAD-Best partners focus on maintaining effective and frequent channels of communication with all alliance partners in this phase. Regular project meetings are essential to evaluate the status of all work, determine the next steps, and monitor the financial resources and compliance with an agreed timeline. Alliance partners receive feedback on their work to ensure high motivation and quality of work. If necessary, they are reminded of their responsibilities.

One key activity is the opening ceremony with all the stakeholders and patrons. Holding a press conference at the opening ceremony is advisable and is a good opportunity to provide local media with a press kit and media guidelines on reporting on suicides. A leaflet about the regional alliance against depression with basic information about depression and a map of contacts where to get help locally and ways to support the alliance (by donating, as a volunteer) should be distributed at the event. Setting up a public newsletter is a good way to promote the alliance and attendees at the opening ceremony can be invited to sign up for it. A range of material templates for the opening ceremony are provided by EAAD.

The main activities during the implementation phase are the recruitment and training of health professionals (level 1) and community facilitators or gatekeepers (level 3). The training can be delivered in person or through videoconferences.

Other activities concern an ongoing involvement with the media and the distribution of informative materials (especially flyers and posters) as a crucial aspect of the public awareness campaign (level 2). Talks and public events should take place continuously during the intervention period, as this not only benefits the dissemination of alliance activities but also indirectly raises awareness about depression as a health problem. Patients and relatives should be included as speakers at the events, as this will help fight stigma.

#### Dissemination of the 4-Level Concept to New Regions in Transfer Countries

In the 3 transfer countries, the goal was to expand the 4-level intervention activities from an existing model project to new regions. To achieve this goal, the following intervention steps were taken:

- Through press releases and other media activities, national and regional health politicians and stakeholders as well as nongovernmental organizations and self-help organizations were informed about the 4-level intervention concept. These activities should raise interest and motivation to start their own local alliances against depression and suicidal behavior in different communities.
- Those who were interested in starting a new regional alliance were supported in several ways: (1) financial support (ie, with a start-up budget of €3.800 [US $4.02]), (2) train-the-trainer sessions, (3) access to EAAD implementation materials and help with their local adaptation, and (4) continuous advice by the EAAD coordination center.
- The existing National Coordination Centre established a national learning and dissemination network of regional alliances by inviting all interested stakeholders (those implementing or interested in implementing regional 4-level interventions) to regular meetings. These allowed us to exchange experiences and best practices but also involved workshops on topics such as fundraising, involving volunteers, or organizing press conferences.

#### Nationwide Uptake of the iFightDepression Tool in All Participating Countries

In order to make the iFightDepression tool available nationwide in all participating countries (ie, implementation and transfer countries), the following intervention steps have been implemented: (1) translation and cultural adaptation of the tool for all countries as needed; (2) in Greece, Italy, Hungary, and Spain, the iFightDepression tool was already implemented, and in all other intervention countries (Estonia, Bulgaria, Poland, and Ireland), a coordination site was appointed to manage the use of the iFightDepression tool; (3) advertised the tool to the general public and health professionals (GPs, MHCPs) through press releases, other media activities, and approaching professional organizations for GPs and MHCPs; and (4) trained health professionals to become guides and give patients access to the tool. The trainings were delivered in person or through videoconferences. e-Learning materials for GPs have been developed that allow them to complete the training at their convenience. It is followed by a test assessing correct understanding.

### Evaluation of the Implementation of the 4-Level Concept in Implementation and Transfer Countries

#### Overview

Our evaluation approach considered monitoring and outcome measures. The monitoring evaluation involved process indicators addressing the extent to which planned activities took place in implementation and transfer countries. It also considered output indicators assessing the quantity and quality of the implemented activities. The outcome evaluation will test to what extent the 4-level intervention generated the expected positive effects in health professionals, community facilitators, and iFightDepression tool users.

Evaluation activities in implementation and transfer countries use the same materials and procedures described below.

#### Monitoring Evaluation

Process and output indicators were collected through a monitoring instrument twice per year. It presented a selection of core indicators of the 4-level intervention activities (eg, occurrence of the opening event and the number of attendees) to track whether planned activities took place and to assess their quantity and quality.

#### Outcome Evaluation

Given the limited time frame of this project, it was not feasible to evaluate whether the intended long-term outcomes of the 4-level concept (ie, reduction of suicidal behavior and improvements in the care of patients with depression) were achieved. Therefore, intermediate outcome indicators that are more directly linked to the operational goals of the 4-level intervention were selected. These are changes in self-judgment of the performance, importance, and competence of treating persons with depression and suicidal ideation. They were measured in health professionals and community facilitators participating in the level-1 and level-3 training sessions using self-developed questionnaires.

Data were collected at 2 measurement time points: before and immediately after the level-1 and -3 trainings. Informed consent was obtained before the first training session.

#### Evaluation of the iFightDepression Tool in All Participating Countries

To evaluate the iFightDepression tool and its effects, users were asked to complete 2 outcome measures. Their informed consent was obtained when registering for the iFightDepression tool, and all data were directly collected within the application. First, after their registration, users were asked to complete a self-developed survey measuring initial expectations regarding the tool. Then, over the following 8 weeks, mental well-being was assessed on a weekly basis with the Patient Health Questionnaire (PHQ-9) [20].

### Ethical Considerations

Participating in any evaluation activity will require informed consent. Ethical approval has been obtained in all implementation and transfer countries before the start of data collection. The names of the ethics committees that approved the study are as follows: (1) Bulgaria: Central Ethics Committee of the Ministry of Health Republic of Bulgaria; (2) Estonia: Tervise Arengu Instituudi inimuuringute eetikakomitee, Research Ethics Committee of the National Institute for Health Development; (3) Greece: Department of Education, Research and Documentation Association of Regional Development and Mental Health; (4) Poland: ethics committee of the Maria Grzegorzewska University; (5) Hungary: Egészségügyi Tudományos Tanács, Tudományos és Kutatásetikai Bizottság; (6) Ireland: Clinical Research Ethics Committee of the Cork Teaching Hospitals; and (7) Spain: Comité Ético de Investigación Clínica de Galicia; Comité Ético de Investigación del Hospital Clínico de Valencia; and Comité de Ética de Investigación de los Hospitales Universitarios Virgen Macarena.

## Results

Data collection was completed in March 2024. Data processing and analyses commenced in the autumn of 2023 and are expected to be completed by the end of 2024. Results are expected to be published in 2025.

## Discussion

### Principal Outcomes

EAAD-Best had three main objectives: (1) implementation of the 4-level intervention in model regions of EU countries in which the intervention had not yet been established, (2) dissemination of the 4-level intervention to new regions in EU countries in which it had already been implemented, and (3) nationwide promotion of the iFightDepression tool in participating countries. In general, all these objectives have been successfully achieved, as alliances against depression were established in all participating countries, iFightDepression guides were trained, and increasing numbers of people started using the tool. Analyses of the collected monitoring data (currently ongoing, results expected to be published in 2025) will allow a more fine-grained conclusion regarding the quantity and quality of intervention activities. In line with previous studies (eg, [8,14]), we anticipate that the outcome evaluation (results expected to be published in 2025) will show that training of GPs, MHCPs, and gatekeepers will increase their subjective competence and their willingness to treat persons with depression and suicidal ideation in the future. Furthermore, iFightDepression tool users are expected to report a decrease in depressive symptoms. The anticipated results will add to the scarce literature on suicide prevention interventions [8] and, most importantly, will extend existing evidence on the effectiveness of the 4-level intervention and the iFightDepression tool to new regions and countries across Europe.

The following sections will discuss some practical issues involved in implementing and evaluating the 4-level concept and the iFightDepression tool and will highlight not only some of the challenges but also the strengths of the approach.

### Participation of Professionals in EAAD Training

Participation in EAAD training sessions is completely voluntary. Professionals will not be remunerated for their participation. Recruiting GPs can be especially challenging due to time constraints and high workloads. Accordingly, trainings focus on practical materials for GPs and their patients, and are delivered in a convenient fashion (eg, in the evenings and online). The introduction of e-learning materials to allow GPs to complete the iFightDepression guide training has been very well received. It has also proven helpful to be able to award Continuing Medical Education points for training completion.

Training professionals will strengthen both their expertise in recognizing mental health issues and their ability to offer support. It is important to stress those benefits in communications. In particular, becoming a guide for the iFightDepression tool enables GPs to provide self-management support based on cognitive behavioral therapy to their patients.

### Potential Risks and Benefits of Using the iFightDepression Tool

Previous studies have shown that the iFightDepression tool has beneficial effects on mental health without causing any adverse effects [14,15]. Therefore, the benefits of participation are expected to outweigh any potential risks (eg, suicidal crises can be overlooked) and unwanted effects (eg, an increase in rumination and somatization due to self-monitoring). In addition, all participants will remain within the usual care of their GPs or MHCPs while using the iFightDepression tool.

The expected benefits of the iFightDepression tool include increased uptake of treatment as online self-help tools are more accessible than traditional forms of psychological support. Hence, the iFightDepression tool has the potential to close the gap between people with depression seeking help and the limited immediate access to psychotherapy [1]. Furthermore, there will be a lower threshold to engage certain people who may be reluctant toward traditional mental health services. Given that the iFightDepression tool is used online and anonymously, users face no risk of stigmatization. The early detection of suicidal ideation and severe symptoms of depression through the inclusion of the PHQ-9 within the tool will help prevent attempted and completed suicides. In particular, the PHQ-9 contains 1 item that directly addresses suicidal ideation. If participants choose a response option indicating that they are experiencing suicidal thoughts or obtain a total score of 15 or above (indicating moderate-to-vigorous symptoms of depression), they will receive an automatic message suggesting contacting their health care professional, GP, or a quality-assured support service. Finally, the tool will strengthen the coping skills of participants, enabling them to manage current and future experiences of depression better.

### Sustainability

The ultimate goal of EAAD-Best is that activities will continue to run and expand from model projects to national networks of alliances against depression upon project completion. In general, sustainability is a key aspect of the 4-level intervention. It has been considered in its development, for example, by making it as cost-effective as possible, flexible, and adaptive to various contexts and encouraging activities leading to an ownership feeling in the communities. Sustainability is further considered within implementation activities by asking partners after a defined intervention phase (2-3 years) to reflect on previous and ongoing activities, their main partners and stakeholders, experienced challenges, and facilitators so on. EAAD further recommends partners to create a national coordinator position that, on the basis of the experiences from the model project, can support and coordinate the dissemination of the approach in further cities and regions to make it efficient, cost-effective, and impactful.

### Evaluation Challenges

Performing applied research like the presented implementation science project can pose challenges such as variability in implementation fidelity. This project tried to minimize such risks by implementing certain measures or processes. In general, questionnaires were always kept to a minimum to avoid overburdening project partners, professionals involved in trainings, and patients using the iFightDepression tool. The dedicated work package running evaluation activities in EAAD-Best provided templates for all assessments and reported completion rates at consortium meetings so that potential difficulties in certain countries could be identified and addressed right away. The training evaluations could be completed online or through paper and pencil to suit the local preferences and increase data completion. To match repeated assessments of training participants, a subject-generated identification code was used to allow correct matching while ensuring anonymity. Future publications on the results of the EAAD-Best project will discuss identified challenges and the chosen solutions in more depth.

### Conclusion

The EAAD-Best project will contribute to improved depression care and prevention of suicidal behavior in Europe by extending the application of the 4-level intervention and the uptake of the iFightDepression tool. Besides supporting the implementation of best practices in mental health promotion and treatment, EAAD-Best will also add to the implementation research evidence base in the area of mental health interventions. Implementing and evaluating a multilevel intervention, such as the 4-level intervention, bears certain risks and challenges, for example, convincing health professionals to join level-1 trainings, establishing wide and consistent networks of stakeholders, and finding funding opportunities to maintain and extend activities upon project completion, and so on. Lessons learned and identified pitfalls will be shared once the monitoring and outcome analyses have been completed (expected by the end of 2024 with resulting publications in 2025), to avoid costly mistakes and ensure an efficient implementation process with a wide and enduring impact beyond the EAAD-Best project in the future.

## Abbreviations

EAAD: European Alliance Against Depression
EU: European Union
GP: general practitioner
MHCP: mental health care professional
PHQ-9: Patient Health Questionnaire

## Data Availability

The datasets generated and analyzed during this study are available from the corresponding author on reasonable request.
